# Supplementary material for: Characterization of the Cardiac Structure and Function of Conscious D2.B10-Dmdmdx/J (D2-mdx) mice from 16–17 to 24–25 Weeks of Age
Source: Int J Mol Sci. 2023 Jul 22;24(14):11805. doi: 10.3390/ijms241411805 (PMC10380312; doi:10.3390/ijms241411805)
Supplement: Supplementary file 1 [file ijms-24-11805-s001.zip › ijms-2500737-supplementary.pptx]

## Slide 1
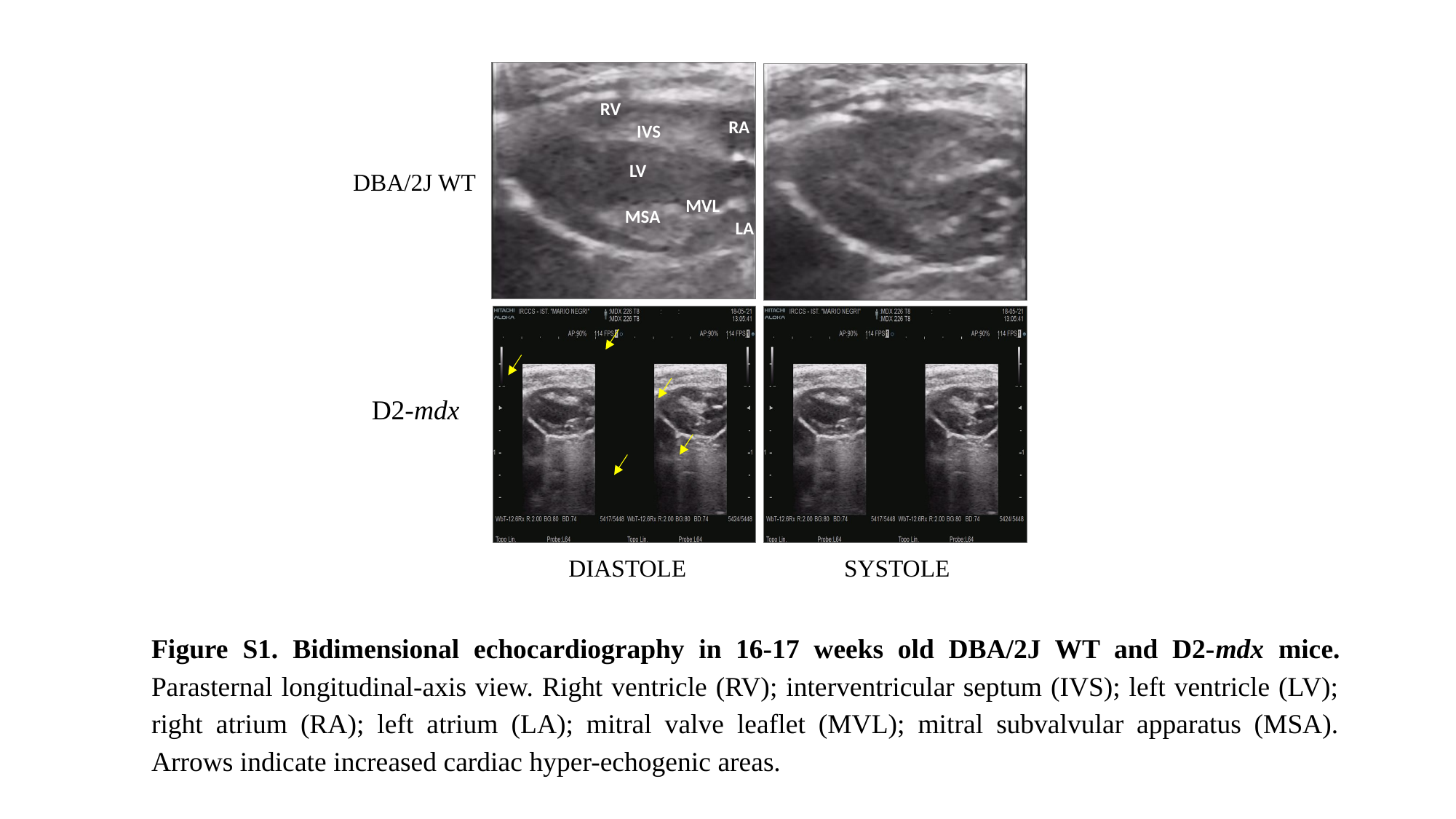

IVS
LV
DBA/2J WT
D2-mdx
DIASTOLE
SYSTOLE
2 mm
RV
RA
MVL
MSA
LA
Figure S1. Bidimensional echocardiography in 16-17 weeks old DBA/2J WT and D2-mdx mice. Parasternal longitudinal-axis view. Right ventricle (RV); interventricular septum (IVS); left ventricle (LV); right atrium (RA); left atrium (LA); mitral valve leaflet (MVL); mitral subvalvular apparatus (MSA). Arrows indicate increased cardiac hyper-echogenic areas.

## Slide 2
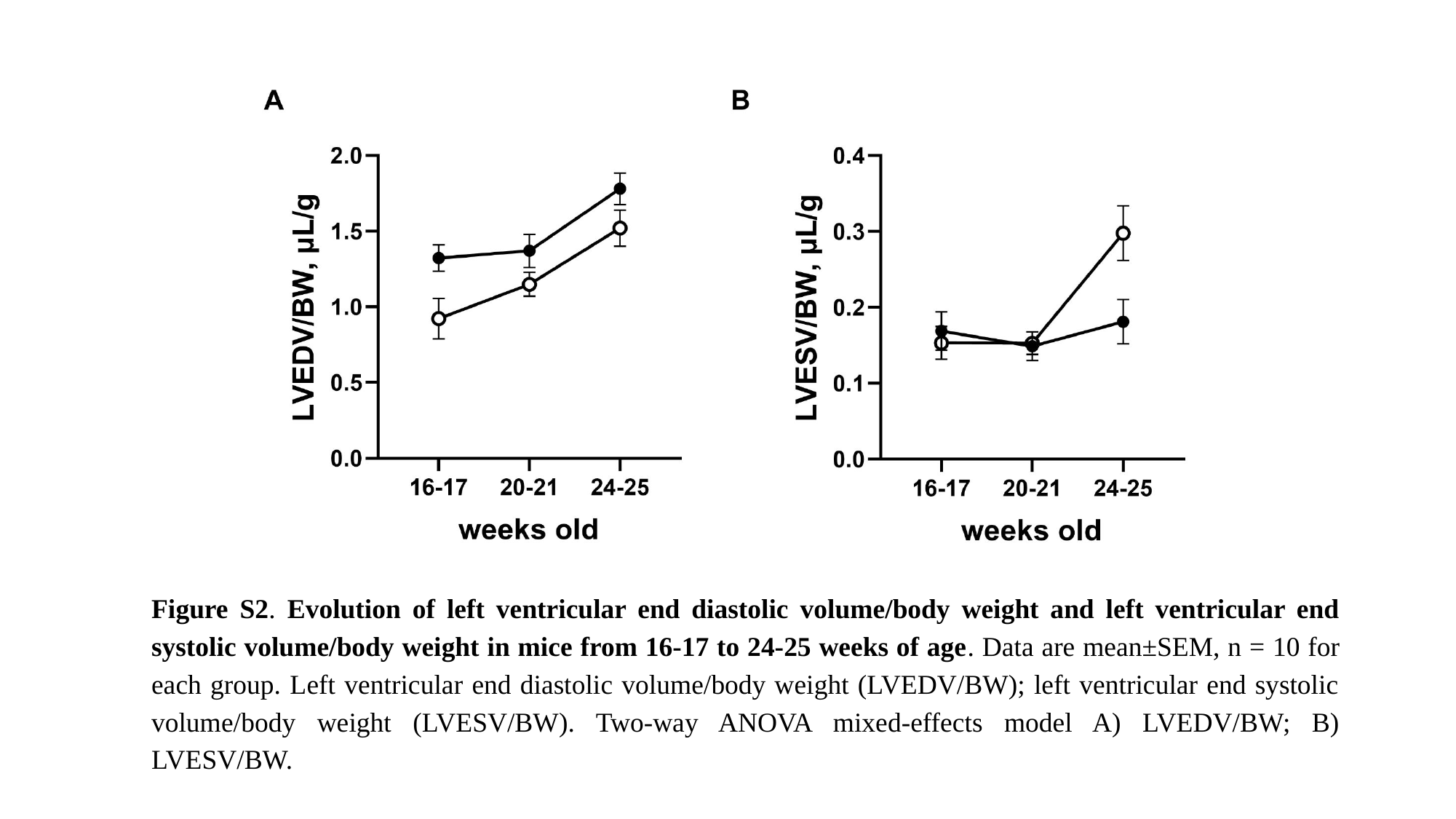

Figure S2. Evolution of left ventricular end diastolic volume/body weight and left ventricular end systolic volume/body weight in mice from 16-17 to 24-25 weeks of age. Data are mean±SEM, n = 10 for each group. Left ventricular end diastolic volume/body weight (LVEDV/BW); left ventricular end systolic volume/body weight (LVESV/BW). Two-way ANOVA mixed-effects model A) LVEDV/BW; B) LVESV/BW.

## Slide 3
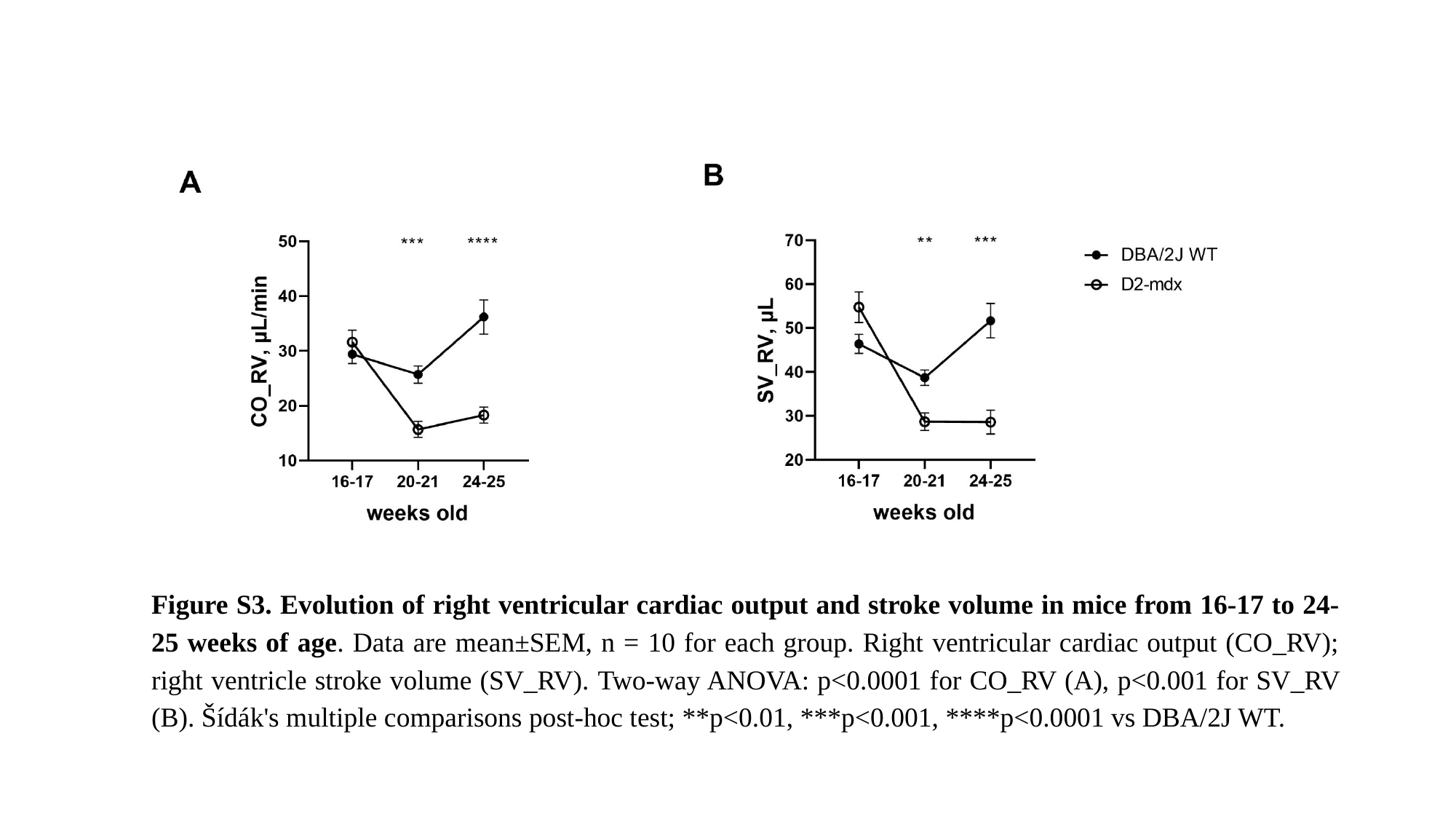

Figure S3. Evolution of right ventricular cardiac output and stroke volume in mice from 16-17 to 24-25 weeks of age. Data are mean±SEM, n = 10 for each group. Right ventricular cardiac output (CO_RV); right ventricle stroke volume (SV_RV). Two-way ANOVA: p<0.0001 for CO_RV (A), p<0.001 for SV_RV (B). Šídák's multiple comparisons post-hoc test; **p<0.01, ***p<0.001, ****p<0.0001 vs DBA/2J WT.

## Slide 4
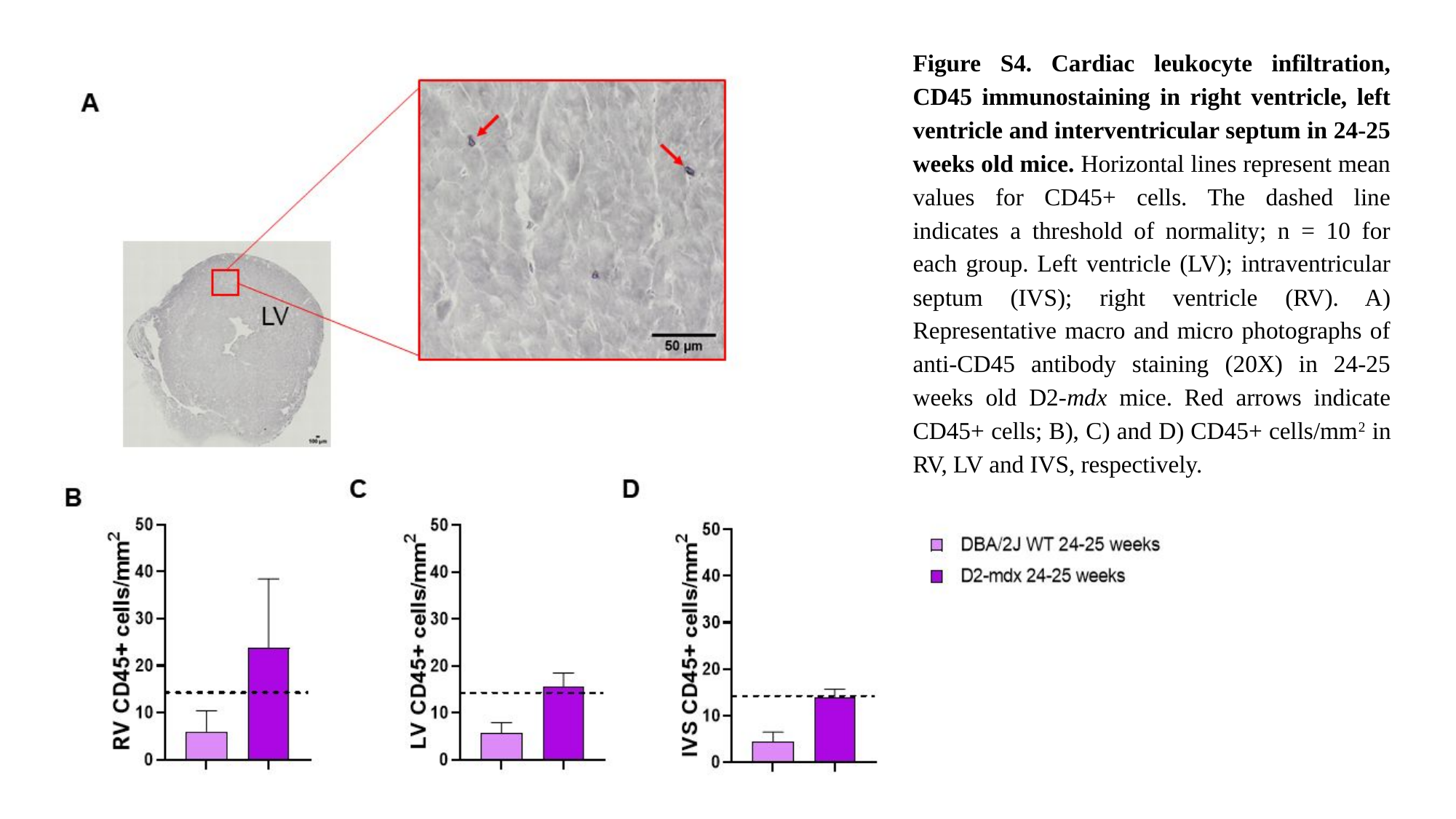

Figure S4. Cardiac leukocyte infiltration, CD45 immunostaining in right ventricle, left ventricle and interventricular septum in 24-25 weeks old mice. Horizontal lines represent mean values for CD45+ cells. The dashed line indicates a threshold of normality; n = 10 for each group. Left ventricle (LV); intraventricular septum (IVS); right ventricle (RV). A) Representative macro and micro photographs of anti-CD45 antibody staining (20X) in 24-25 weeks old D2-mdx mice. Red arrows indicate CD45+ cells; B), C) and D) CD45+ cells/mm2 in RV, LV and IVS, respectively.

## Slide 5
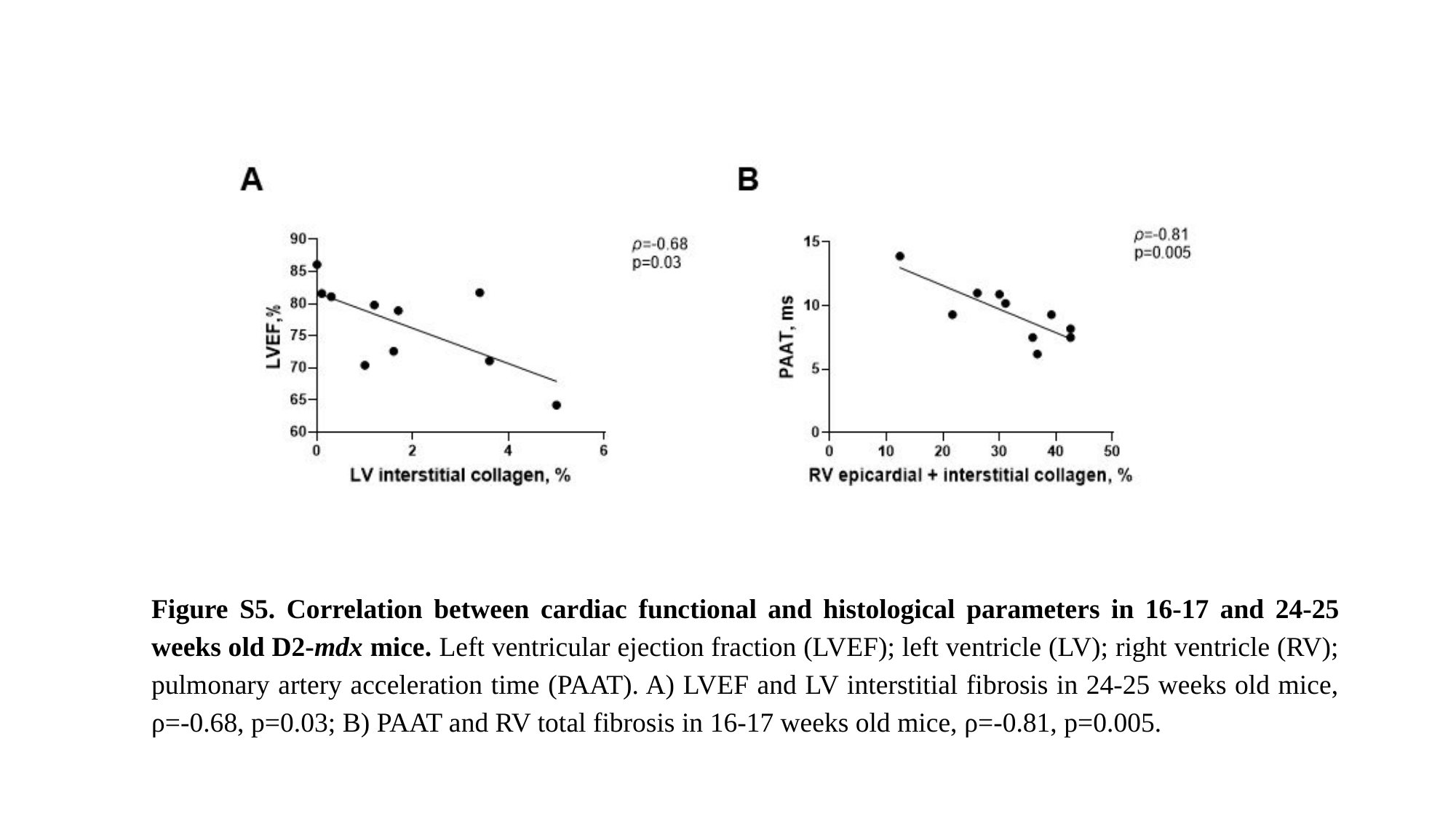

Figure S5. Correlation between cardiac functional and histological parameters in 16-17 and 24-25 weeks old D2-mdx mice. Left ventricular ejection fraction (LVEF); left ventricle (LV); right ventricle (RV); pulmonary artery acceleration time (PAAT). A) LVEF and LV interstitial fibrosis in 24-25 weeks old mice, ρ=-0.68, p=0.03; B) PAAT and RV total fibrosis in 16-17 weeks old mice, ρ=-0.81, p=0.005.

## Slide 6
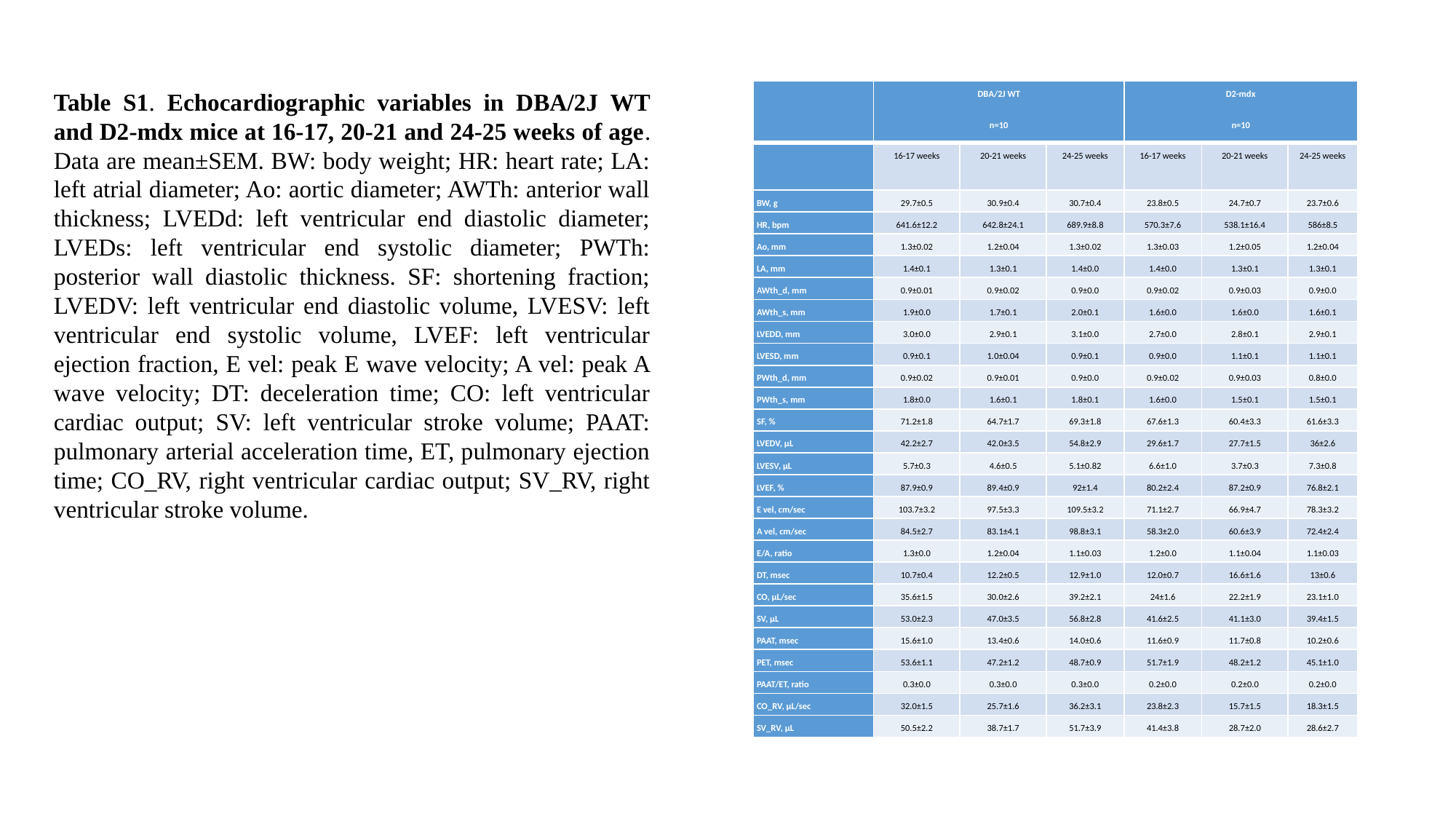

Table S1. Echocardiographic variables in DBA/2J WT and D2-mdx mice at 16-17, 20-21 and 24-25 weeks of age. Data are mean±SEM. BW: body weight; HR: heart rate; LA: left atrial diameter; Ao: aortic diameter; AWTh: anterior wall thickness; LVEDd: left ventricular end diastolic diameter; LVEDs: left ventricular end systolic diameter; PWTh: posterior wall diastolic thickness. SF: shortening fraction; LVEDV: left ventricular end diastolic volume, LVESV: left ventricular end systolic volume, LVEF: left ventricular ejection fraction, E vel: peak E wave velocity; A vel: peak A wave velocity; DT: deceleration time; CO: left ventricular cardiac output; SV: left ventricular stroke volume; PAAT: pulmonary arterial acceleration time, ET, pulmonary ejection time; CO_RV, right ventricular cardiac output; SV_RV, right ventricular stroke volume.
| | DBA/2J WT n=10 | | | D2-mdx n=10 | | |
| --- | --- | --- | --- | --- | --- | --- |
| | 16-17 weeks | 20-21 weeks | 24-25 weeks | 16-17 weeks | 20-21 weeks | 24-25 weeks |
| BW, g | 29.7±0.5 | 30.9±0.4 | 30.7±0.4 | 23.8±0.5 | 24.7±0.7 | 23.7±0.6 |
| HR, bpm | 641.6±12.2 | 642.8±24.1 | 689.9±8.8 | 570.3±7.6 | 538.1±16.4 | 586±8.5 |
| Ao, mm | 1.3±0.02 | 1.2±0.04 | 1.3±0.02 | 1.3±0.03 | 1.2±0.05 | 1.2±0.04 |
| LA, mm | 1.4±0.1 | 1.3±0.1 | 1.4±0.0 | 1.4±0.0 | 1.3±0.1 | 1.3±0.1 |
| AWth\_d, mm | 0.9±0.01 | 0.9±0.02 | 0.9±0.0 | 0.9±0.02 | 0.9±0.03 | 0.9±0.0 |
| AWth\_s, mm | 1.9±0.0 | 1.7±0.1 | 2.0±0.1 | 1.6±0.0 | 1.6±0.0 | 1.6±0.1 |
| LVEDD, mm | 3.0±0.0 | 2.9±0.1 | 3.1±0.0 | 2.7±0.0 | 2.8±0.1 | 2.9±0.1 |
| LVESD, mm | 0.9±0.1 | 1.0±0.04 | 0.9±0.1 | 0.9±0.0 | 1.1±0.1 | 1.1±0.1 |
| PWth\_d, mm | 0.9±0.02 | 0.9±0.01 | 0.9±0.0 | 0.9±0.02 | 0.9±0.03 | 0.8±0.0 |
| PWth\_s, mm | 1.8±0.0 | 1.6±0.1 | 1.8±0.1 | 1.6±0.0 | 1.5±0.1 | 1.5±0.1 |
| SF, % | 71.2±1.8 | 64.7±1.7 | 69.3±1.8 | 67.6±1.3 | 60.4±3.3 | 61.6±3.3 |
| LVEDV, µL | 42.2±2.7 | 42.0±3.5 | 54.8±2.9 | 29.6±1.7 | 27.7±1.5 | 36±2.6 |
| LVESV, µL | 5.7±0.3 | 4.6±0.5 | 5.1±0.82 | 6.6±1.0 | 3.7±0.3 | 7.3±0.8 |
| LVEF, % | 87.9±0.9 | 89.4±0.9 | 92±1.4 | 80.2±2.4 | 87.2±0.9 | 76.8±2.1 |
| E vel, cm/sec | 103.7±3.2 | 97.5±3.3 | 109.5±3.2 | 71.1±2.7 | 66.9±4.7 | 78.3±3.2 |
| A vel, cm/sec | 84.5±2.7 | 83.1±4.1 | 98.8±3.1 | 58.3±2.0 | 60.6±3.9 | 72.4±2.4 |
| E/A, ratio | 1.3±0.0 | 1.2±0.04 | 1.1±0.03 | 1.2±0.0 | 1.1±0.04 | 1.1±0.03 |
| DT, msec | 10.7±0.4 | 12.2±0.5 | 12.9±1.0 | 12.0±0.7 | 16.6±1.6 | 13±0.6 |
| CO, µL/sec | 35.6±1.5 | 30.0±2.6 | 39.2±2.1 | 24±1.6 | 22.2±1.9 | 23.1±1.0 |
| SV, µL | 53.0±2.3 | 47.0±3.5 | 56.8±2.8 | 41.6±2.5 | 41.1±3.0 | 39.4±1.5 |
| PAAT, msec | 15.6±1.0 | 13.4±0.6 | 14.0±0.6 | 11.6±0.9 | 11.7±0.8 | 10.2±0.6 |
| PET, msec | 53.6±1.1 | 47.2±1.2 | 48.7±0.9 | 51.7±1.9 | 48.2±1.2 | 45.1±1.0 |
| PAAT/ET, ratio | 0.3±0.0 | 0.3±0.0 | 0.3±0.0 | 0.2±0.0 | 0.2±0.0 | 0.2±0.0 |
| CO\_RV, µL/sec | 32.0±1.5 | 25.7±1.6 | 36.2±3.1 | 23.8±2.3 | 15.7±1.5 | 18.3±1.5 |
| SV\_RV, µL | 50.5±2.2 | 38.7±1.7 | 51.7±3.9 | 41.4±3.8 | 28.7±2.0 | 28.6±2.7 |

## Slide 7
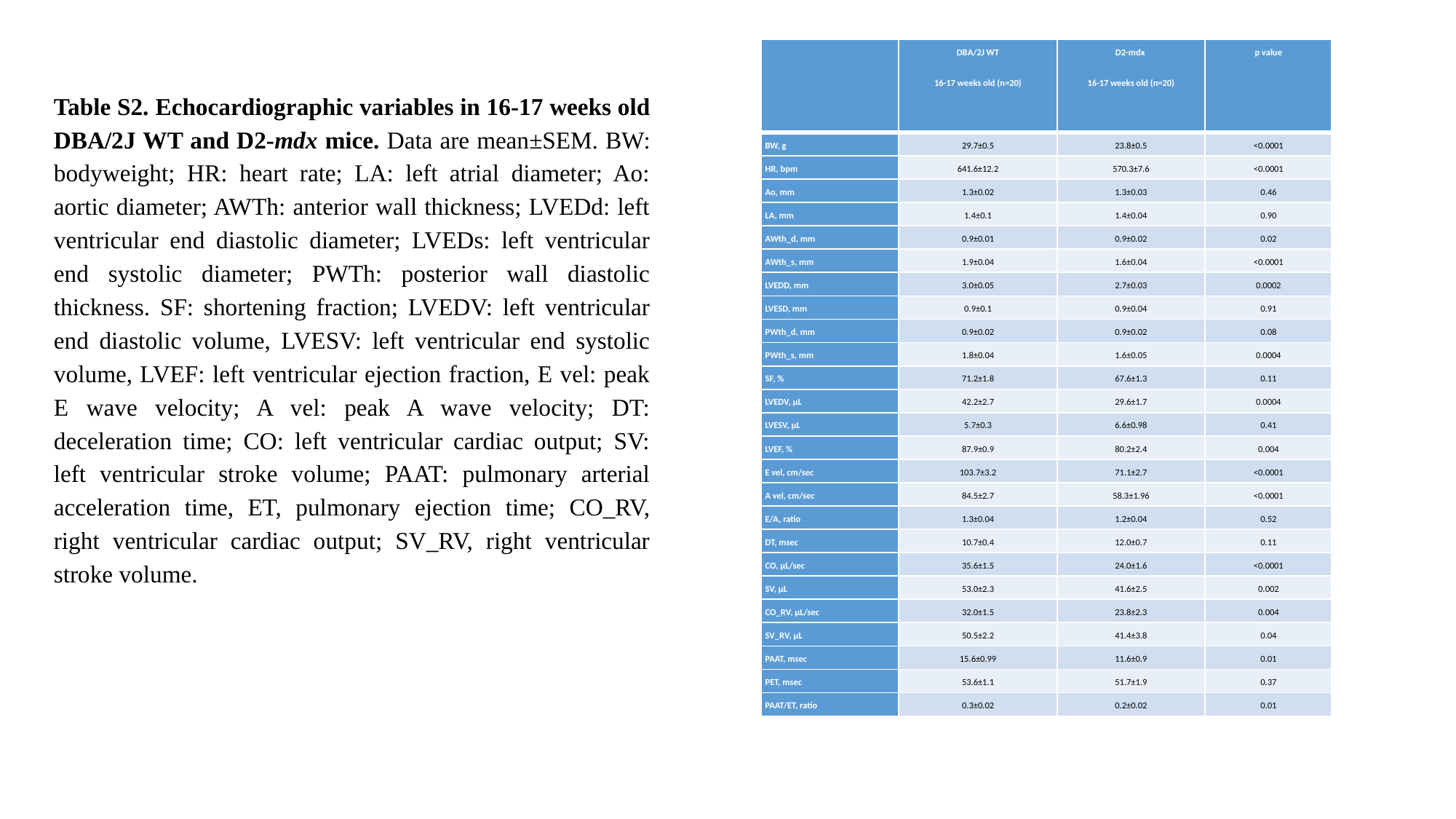

| | DBA/2J WT 16-17 weeks old (n=20) | D2-mdx 16-17 weeks old (n=20) | p value |
| --- | --- | --- | --- |
| BW, g | 29.7±0.5 | 23.8±0.5 | <0.0001 |
| HR, bpm | 641.6±12.2 | 570.3±7.6 | <0.0001 |
| Ao, mm | 1.3±0.02 | 1.3±0.03 | 0.46 |
| LA, mm | 1.4±0.1 | 1.4±0.04 | 0.90 |
| AWth\_d, mm | 0.9±0.01 | 0.9±0.02 | 0.02 |
| AWth\_s, mm | 1.9±0.04 | 1.6±0.04 | <0.0001 |
| LVEDD, mm | 3.0±0.05 | 2.7±0.03 | 0.0002 |
| LVESD, mm | 0.9±0.1 | 0.9±0.04 | 0.91 |
| PWth\_d, mm | 0.9±0.02 | 0.9±0.02 | 0.08 |
| PWth\_s, mm | 1.8±0.04 | 1.6±0.05 | 0.0004 |
| SF, % | 71.2±1.8 | 67.6±1.3 | 0.11 |
| LVEDV, µL | 42.2±2.7 | 29.6±1.7 | 0.0004 |
| LVESV, µL | 5.7±0.3 | 6.6±0.98 | 0.41 |
| LVEF, % | 87.9±0.9 | 80.2±2.4 | 0.004 |
| E vel, cm/sec | 103.7±3.2 | 71.1±2.7 | <0.0001 |
| A vel, cm/sec | 84.5±2.7 | 58.3±1.96 | <0.0001 |
| E/A, ratio | 1.3±0.04 | 1.2±0.04 | 0.52 |
| DT, msec | 10.7±0.4 | 12.0±0.7 | 0.11 |
| CO, µL/sec | 35.6±1.5 | 24.0±1.6 | <0.0001 |
| SV, µL | 53.0±2.3 | 41.6±2.5 | 0.002 |
| CO\_RV, µL/sec | 32.0±1.5 | 23.8±2.3 | 0.004 |
| SV\_RV, µL | 50.5±2.2 | 41.4±3.8 | 0.04 |
| PAAT, msec | 15.6±0.99 | 11.6±0.9 | 0.01 |
| PET, msec | 53.6±1.1 | 51.7±1.9 | 0.37 |
| PAAT/ET, ratio | 0.3±0.02 | 0.2±0.02 | 0.01 |
Table S2. Echocardiographic variables in 16-17 weeks old DBA/2J WT and D2-mdx mice. Data are mean±SEM. BW: bodyweight; HR: heart rate; LA: left atrial diameter; Ao: aortic diameter; AWTh: anterior wall thickness; LVEDd: left ventricular end diastolic diameter; LVEDs: left ventricular end systolic diameter; PWTh: posterior wall diastolic thickness. SF: shortening fraction; LVEDV: left ventricular end diastolic volume, LVESV: left ventricular end systolic volume, LVEF: left ventricular ejection fraction, E vel: peak E wave velocity; A vel: peak A wave velocity; DT: deceleration time; CO: left ventricular cardiac output; SV: left ventricular stroke volume; PAAT: pulmonary arterial acceleration time, ET, pulmonary ejection time; CO_RV, right ventricular cardiac output; SV_RV, right ventricular stroke volume.
